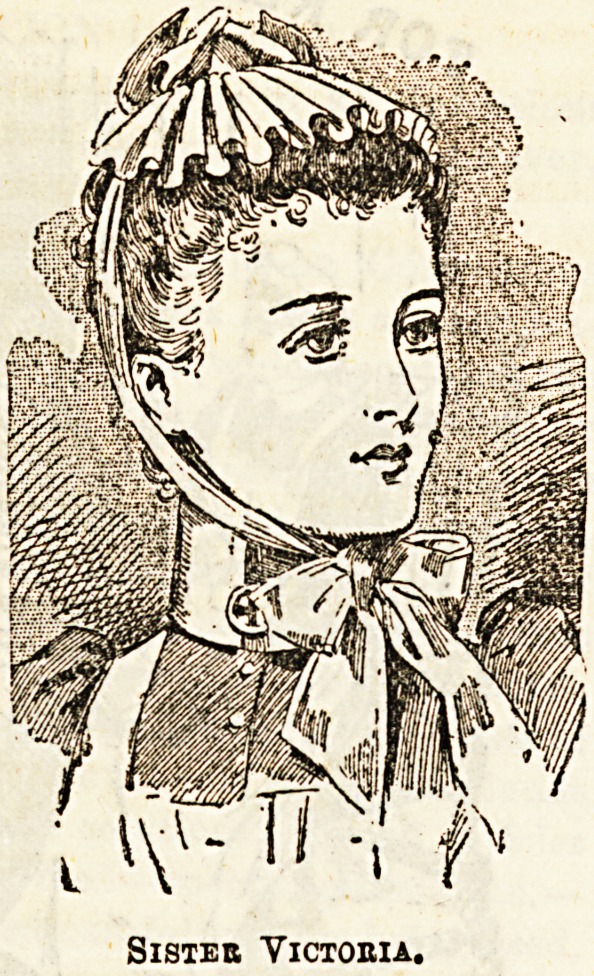# The Hospital Nursing Supplement

**Published:** 1892-06-18

**Authors:** 


### "The Hospital" Nursing Mirror.

Being the Extra Nursing Supplement of "The Hospital" Newspaper.

Contributions for this Supplement should be addressed to the Editor, The Hospital, 140, Strand, London, W.C., and should have the word "Nursing" plainly written in left-hand top corner of the envelope.

### En Passant.

EDINBURGH ROYAL INFIRMARY.—The two days' sale to provide a few extra luxuries for this nursing home was very successful. The Marquis of Tweeddale opened the sale, which realised £450. A library, a few easy chairs, pictures, and so on will now be added to the nurses' home.

NURSES' CO-OPERATION.—The meeting of the Executive Committee of the Nurses Co-operation took place on the 8th inst., and a great deal of business was satisfactorily transacted. We are glad to find the association continues to grow and prosper and, we hear, that it has already more than fulfilled the expectations of the promoters of the scheme.

A TRAINED NURSE FOR CASTOR.—A nurse is to start nursing in Castor in August, and the Rev. W. Hopkinson has generously offered to hold himself responsible for the first year's expenses, by which time he feels sure the public will have thoroughly realised the benefit a sick nurse confers, and will have come forward with subscriptions. We commend this novel view to the consideration of others.

BEDFORDSHIRE NURSES' INSTITUTE. — The bazaar at the Bedford Corn Exchange on June 1st was an undoubted success. Money was needed to provide more accommodation for the nursing staff and to provide additional funds for the district nursing. Mrs. Rawson, the Lady Superintendent, the local branch of the St. John Ambulance Association, and Mrs. Alfred Paine initiated the undertaking, which was very well supported by the residents in the neighbourhood.

WANTS AND WORKERS.—We have been much interested in watching the steady advance made by our column of Wants and Workers, which started just over a year ago, for it has shown us that it supplies a need, and it is also very gratifying to find how very seldom we ask for anything in our columns which our readers do not provide us with. No appeal is inserted until we have satisfied ourselves that it is not only a genuine but a necessary one. There are plenty of gaps that want filling, and there are plenty of workers who can fill them, and is it not a kindness after all to fill up a perhaps somewhat empty life with something to do?

SHORT ITEMS.—The second year's report of the Warwick District Nursing tells of much good done, and of 3,480 visits paid during the past year by Nurse Scott.—The Lady-in-Charge of the Helena Nursing Home, Reading, which receives twelve homeless ladies, suffering from incurable diseases, appeals for more funds that the work may not flag.—The Hampshire Nurses' Institute is considering the question of providing fully-trained nurses for their district nursing branch, and of affiliating with the Q.V.J.I.N.; a public meeting at Southampton is to decide the question.—The Keighley Board of Guardians are a striking example of modern progress; they do not consider a night nurse necessary for the Infirmary; perhaps the Local Government Board memorandum has not come to their notice.—A Nurses' Home is to be built as an adjunct to the infirmary at Carlisle; it will form an upper floor to the dispensary. All the nurses who went to Miss Parmenter's, at Russell's Farm, Wethersfield, near Braintree, last year, are, with one exception, going there again this summer, and there is room for a few others.

EDINBURGH LADY MEDICAL STUDENTS.—An application has been made by the Scottish Association for the Medical Education of Women to the Managers of the Edinburgh Royal Infirmary requesting that arrangements be made for the clinical instruction of twelve women. The managers, who are anxious to accede to the request, consulted the staff, but at a meeting it was decided, by fifteen votes to five, that until the infirmary extension was carried out the arrangements could not be made. It is said that the managers are not satisfied with this reply, and we hope soon to see the women students gain their point.

OPENING OF NEW WARDS AT COLD ASH COTTAGE HOSPITAL.—The opening of new wards at Miss Bowditch's Cottage Hospital, Cold Ash, Newbury, occasioned much rejoicing on the 8th inst., when a large party assembled, 85 guests being entertained at a luncheon, for which most of the good things were sent by friends. Twenty cots are now added to the original fifteen, in which so many sick children have been nursed back to life and health, and Miss Bowditch has accommodation for a few private patients, for whom special arrangements and terms are made. For children occupying cots in the wards the charge is 7s. each per week.

TEA AND TEA DRINKING.—A quiet place for that necessity of a nurse's life, a cup of tea, has discovered itself to us in "The Ladies' Own" Tea Association at 92, New Bond Street, where, in the pleasantest and shadiest of rooms, a refreshing meal is provided at a reasonable price. The lady tea dealers have done an excellent work in opening a fresh path for enterprise of their own sex, who will doubtless avail themselves of so good a sphere for their abilities, whilst it is comparatively fresh and free. Presently, like all fields for women's work, the supply will probably exceed the demand for lady labourers in the tea trade.

A WARNING TO NURSES.—We have a letter from a correspondent in Paris strongly urging English nurses not to be deluded into going to any so-called nurses homes in that city, whether kept by English or French women, unless they have proof of their being honest and genuine establishments. Our correspondent thinks that a nurses' hotel (or residential club?) would answer extremely well, but we should think it most desirable that full enquiries should be made by any one willing to start such a scheme, first, as to the need for it, and secondly as to the prospect of its receiving countenance and substantial support by those English already settled in the French capital.

TRAINS AND TRAINED NURSES.—A very animated meeting was held in Berlin the other day, of which an amusing description was given in the *Daily News* of 7th inst. Some two hundred ladies met to discuss the "train" nuisance and to advocate its abolition; they even passed a resolution asking the police to forbid the wearing of trailing skirts in the streets. With this spirited proceeding before us, it is sad to note that the fashion in England is hourly gaining ground, and our frivolous sisters are being initiated by those who ought to know better, viz., trained nurses who continue to wear in the streets uniform dresses sufficiently long to annex the abominations of the pavement. We challenge any observant person who walks along Oxford Street to say whether quite half the nurses she meets do not thus perseveringly aid the scavengers at the expense of their patients.

### Ventilation, Disinfection, and Diet.

By P. Caldwell Smith, M.D.

### X.—DISINFECTION (*continued*).

General rules for Disinfection applicable to all Infectious Diseases—Disinfection in Small-pox, Scarlet Fever, Measles.

Disinfection in the Different Zymotic or Contagious Diseases.—There are certain precautions which are applicable to all these diseases. The excreta which contains the germs of the disease should be in every case destroyed or thoroughly disinfected, and all articles brought into contact and soiled with these, as linen, towels, and handkerchiefs, should be treated in the same way. Except in the case of cholera, where drying has been shown to be an effective means of killing the bacillus, all these articles should be kept moist, preventing the germs from being sown abroad by currents of air. Those in attendance on the patient should wash their hands frequently in sublimate or creolin solutions, and all articles by which the patient has taken food—spoons, glasses, &c.—should be disinfected and cleaned carefully after being used. This may be done by allowing them to stand in creolin solution for a few hours, and then washing in boiling water slightly cooled. I may warn you that pewter, German silver, nickel, or steel articles should not be dipped in or washed with corrosive sublimate, as a deposit of mercury is formed on these, which is difficult to clean off. The nurse should always have a room near the sick room, all the better if it enters directly off it, in which she can change her clothes before going outside, and her meals should always be served in that apartment. Never, when nursing cases of an infectious nature, and especially in cases of typhoid fever and cholera, sit down to a meal without first using the nail brush well, with a strong disinfectant solution, as the sublimate or creolin one. I am decidedly of opinion that when nurses contract typhoid, when nursing a patient suffering from it, they do so from neglecting this precaution; that is, of course, if the disease is not caused in the first instance by some defect in the drainage of the house, in which case, if this is not remedied, the nurse is as liable to take it as was her patient.

In the case of small-pox, no nurse should, of course, be allowed to take charge of a case of this disease unless she has been recently re-vaccinated, or has previously had the disease. All discharges from the mouth and nose should be removed by means of pieces of surgeons' lint, which can at once be burned. The excreta should be received into an utensil in which a strong solution of creolin or carbolic acid is kept, as the sublimate solution is not satisfactory for reasons previously stated, although if a 1 per cent. solution of sublimate be used, disinfection will be complete. These should then be allowed to stand for six or eight hours before being put down the soil-pipe, and when this is done the pipes should be well flushed. It is, of course, necessary that a set of cups, saucers, drinking mugs, and spoons be set apart strictly for the patient's use. As soon as the medical attendant permits, the body of the patient should be smeared with an oily substance, and this is more satisfactory if a disinfectant is mixed with the oil. For this purpose either lano-creolin may be used, or a creolin ointment of the strength of 1 to 8 of ordinary lard. When convalescent, the patient should take a hot bath daily, and the water for the bath should contain a disinfectant, as corrosive sublimate or creolin. These baths should be continued for two or three days after every scab has come off, and then the patient can be allowed to put on clothes, either new ones, or his old ones disinfected, before mixing with other people. One month from the first appearance of the eruption is about the time a patient suffering from this disease has to be isolated.

The above measures are also necessary in scarlet fever, six weeks being about the time of isolation, or until desquamation is complete. Immersion and baths are also necessary, and all discharges from ears, &c., which often continue after so-called convalescence, should be carefully watched, these discharges also being thought by some authorities to be a means of conveying infection.

(*To be continued.*)

### Where to Go.

Trained Nurses' Club.—A lecture will be given at the Midwives' Institute and Trained Nurses' Club, on Friday evening, June 17th, at 8 o'clock, by Mr. Dundas Grant, M.A., M.D., F.R.C.S.Eng., Surgeon to Central Throat and Ear Hospital. Subject: "The Nurses Duty in Diseases of the Throat, including Tracheotomy." A few tickets to non-members 6d. each.

St. James's Hall.—A correspondent thinks nurses may care to know of the West Central London Mission Services, held at St. James's Hall, on Sunday evenings. They begin at half-past six with an orchestral performance. Seats are reserved for nurses in uniform.

### Presentations.

Glasgow Samaritan Hospital for Women.—At a meeting of the directors on Thursday, 2nd inst., Ex-Provost Morrison, on behalf of the directorate, presented Miss C. Robertson, the Matron, with a handsome tea and coffee service on the occasion of her approaching marriage. With the service, Miss Robertson received a copy of the previous monthly meeting minutes, in which the directors expressed their regret at her resignation, and their high appreciation of the manner in which she had discharged the duties of her office to the hospital. On Friday afternoon, the 3rd inst., Professor D. N. Knox, the consulting surgeon to the hospital, on behalf of the medical staff, presented Miss Robertson with a handsome marble timepiece. The meeting was largely attended by the nursing and medical staffs, and Miss Robertson received many congratulations. Miss Robertson was also the recipient of a handsome china tea set from her nursing staff. Miss Robertson served her "apprenticeship" in the wards of the Glasgow Royal Infirmary more especially under Dr. William McEwan. During her tenure of office in the "Samaritan," she has done much to improve the condition of the hospital. She is succeeded in office by Miss Morton, who has been selected from a large number of candidates for the post.

### Notes and Queries.

To Correspondents.—1. Questions or answers may be written on post-cards. 2. Advertisements in disguise are inadmissible. 3. In answering a query please quote the number. 4. A private answer can only be sent in urgent cases, and then a stamped addressed envelope must be enclosed. 5. Every communication must be accompanied by the writer's full name and address, not necessarily for publication. 6. Correspondents are requested to help their fellow nurses by answering such queries as they can.

### Query.

Will anybody kindly give me any information as to whether there is any country or seaside "Home" for poor incurable paralytics, where they could have a change of air for a short time? The case I know of is a very sad one, and such a Home would be a great blessing.

### Answers.

*C. E.* is thanked for the kind subscription of 5s. towards the fund for Mr. Simpkins.

*Clontarf.*—Apply to the Army Medical Department, Victoria Street. Three years' training in some large general hospital is necessary.

*K. P., Sutton.*—One year's general training would be very useful. At Queen Charlotte's Hospital, Marylebone Road, you would pay ten guineas for eight weeks' training, and would gain a certificate. Do you wish to become a midwife and get the London Obstetrical Society's diploma? Write a little more fully to "Nursing" at this office, when we will answer any other questions.

*F. N.*—It all depends; we know a nurse who is not 5 ft. who is described by her Matron as "one of my best nurses;" but why do you not write fully and say why you ask the question? we can then help you much better.

*L.*—If you write to Dr. Shuttleworth, Royal Albert Asylum for the Northern Counties, Lancaster, you will find what you require. Pay patients are received, but you do not mention this point.

### Nursing Homes.

### VI.—LONDON HOSPITAL.

THE nurses' home attached to this hospital has the advantage of being a modern building, with the newest sanitary and other arrangements, including an outside iron staircase, available in case of fire. Every nurse and probationer has a separate bed-room, and there are two bath-rooms on each floor, and also a kind of recess where, over a gas-stove, boiling water is always ready for the stray cup of tea or cocoa so necessary to the comfort of nurses. A small sink carries away superfluous water, and a covered pail beneath it receives tea-leaves, dead flowers, or other discarded rubbish, which is thus conveniently collected and carried away. In a little side corridor there is a stand of numbered pigeon-holes, in which boots requiring brushing are placed, and they are thence conveyed to the basement to be cleaned by the boy specially employed for this purpose. The bed-rooms have stained floors, spring beds, neat chests of drawers, and hanging cupboards with looking-glass doors, these last specially made to fit an angle of the wall and thus save floor space. Standing in one of these home-like little nests, which commands a pleasant view of green grass and trees, we may well exclaim doubtfully, "Can this be Whitechapel?" All the passages are warmed by means of hot-water pipes, and there is a "lift" for luggage, and when the nurses have transferred the contents of their boxes to their drawers, the empty trunks are removed and placed in orderly array in the large attics which lie under the nursing home roof. A special kitchen and staff of servants are also maintained for the nurses, with fitting offices in the basement. There is a large airy dining-room on the first floor, where nurses and probationers take their meals in detachments, and a smaller room adjoining it is used for the Sisters. A "sick-room" for the nurses is upstairs, and it has the comfortable appearance of a kind of private ward. On the floor between it and the dining hall, the nurses' big sitting-room is conveniently placed. It is furnished with sofas and easy chairs, various tables, an open book-case with literature of all sorts, besides daily and weekly papers; and against one wall stands a long, narrow cupboard, whose open doors reveal a valuable collection of beautifully prepared "specimens" presented to the nurses by Mr. F. Treves, F.R.C.S., and Mr. T. H. Openshaw, F.R.C.S. In the dining-room is a closed book-case containing medical works, which are given out at stated times to those applicants who desire to study them. The weekly lecture to probationers, given by a member of the visiting staff, is held in the new clinical theatre, and classes for supplementary instruction in theoretical nursing, are given by some of the Sisters. To appreciate these good and practical arrangements thoroughly, we have only to go back, in thought, to a visit paid some ten years ago, when no nurse had a room to herself, but on the contrary, in some cases three, in others five, and in one room ten adult women were quartered, with only light screens to give any privacy, and no possible chance of securing the peace and quiet which are equally necessary for weary workers. Many other arrangements of that period were in harmony with the sleeping accommodation, and it does not need great intelligence to realise the difference in the aspect of matters to-day, for the place tells its own tale. All honour to those who have combined to make this one of the most complete and comfortable, as well as by far the largest nursing home in London. We must not omit to mention that the night nurses are as well lodged as the day, and that their comfortably-furnished little rooms are situated in the main building of the hospital over some of the wards.

### CONTENTMENT.

A GREAT thinker and a really wise man says, "Contentment is a pearl of great price, and whoever procures it, at the expense of a thousand desires, makes a wise man and a happy purchase." This has been considered the truth by most philosophers and divines, though perhaps it is a little hard to understand; all of us, however, can take in the homely proverbs, "The contented mind is a continual feast," and "What can't be cured, must be endured," both of which mean the same thing.

When a person in health is constantly dissatisfied, grumbling at this thing and that, and wishing everything different, we feel angry, and set him down for a foolish, almost a wicked person thus to spoil his own happiness. But the fretfulness of a sick person gains our sympathy and compassion; we are so sorry for him that we long to give him that even state of mind called contentment, which smooths our pillows, which makes our agonies grow less, our peace of mind greater. It is when we are recovering from an illness that we have most need of this grace. We have been too sick and miserable before to realise anything much about us, now we are better, with the hope of soon regaining complete health, we want to do too much; we chafe at being still kept in bed; we know we could walk about if we were allowed; then we get low spirited, and are sure we shall always be poor creatures in the future. Now at such times it is wise to dismiss from our minds, as far as we can, all thoughts about ourselves, and not be over eager for health or over anxious, lest it should delay its coming, to dwell, in fact, on the brighter prospect opening before us of happiness with wife or husband and child and leave all else besides. This is a difficult thing to do, we all know that, and some give up trying altogether; but we will go to the root of the matter and find out whether we can't have this "pearl" for our own pleasure and adornment. We read in the Bible that "Godliness with contentment is great gain," and "Having food and raiment let us be therewith content." This is what St. Paul tells his young friend Timothy; but he also practised what he preached, for he says in another place, "I have learned in what ever state I am therewith to be content." Happy man! Then he goes on to tell the secret of his success, "I can do all things through Christ which strengtheneth me." He gives up trying by himself, and rests his spirit in God, knowing that all we receive comes from His hand, and as He is perfect in wisdom, pleasure or pain are alike sent for our good. We will pray then to be humble in heart; we will set our love on Christ and desire neither life nor death, but as He wills. "Let our Master do what seemeth Him good." This is the root from which true contentment springs. We will plant this root in our own hearts; water it with tears of sorrow for past discontent; force it with the heat of love, and train it with good resolutions and practical piety.

### Nurses' Outfits.

Sister Mabel.

Sister Victoria.

When a nurse has notice that she is accepted at a hospital, she is informed, at the same time, that she must be prepared to wear a certain uniform, and her facilities for supplying herself with this outfit are greatly increased by the fact that various large drapery establishments have given particular attention to nurses' costumes. For some years uniform cloaks and bonnets, having been recognised as necessities, could be obtained at many shops, "made to order," but a later development has provided "special departments" devoted to catering for the wants of the large and ever-increasing nursing world. The latest establishment visited by us is that of Messrs. Garrould, Edgware-road, where every article of uniform can be supplied in a few days; in fact, a private nurse, working on her own account, and therefore at liberty to choose her own costume, could be supplied at a moment's notice with all she requires. A large stock of aprons is always on hand, in two qualities, one of cotton, linen-faced, full-sized as regards length and breadth, at 2s. 6d. each; and another kind, made of strong, serviceable linen, at 3s. 11d., with square bibs and straps to cross behind and button on to the waistband, and stout, plain cotton dresses, at 15s. each, can be obtained ready made. Every other variety of nurses' gowns, to supply the requirements of hospital rules or private fancy, can be made at short notice. Perhaps one of the chief values of this kind of establishment lies in the fact that an estimate can be obtained before ordering, thus enabling a nurse to judge exactly whether the cost of a complete outfit lies within her means, and whether she can indulge in the comfort of having all trouble in the matter taken off her hands.

Ready-made cloaks of thick or thin cloth, in two shapes, of which "the Russian" is perhaps the most becoming to ordinary figures, are in many colours, with bonnets to match, of blue, grey, or black; the prices of the latter run from 8s. 11d. upwards, but the one at 10s. is a very fair sample of what a nurse's bonnet should be.

The Belmont is a really pretty as well as a nurse-like cap, and is quite as becoming without the hanging strings, which some practical workers object to, especially now that they are so universally adopted by the domestic servant, who has long ago shown by imitation (that sincerest flattery) her appreciation of the merits of the professional apron; the Sister Victoria and Sister Mabel caps are also very pretty and cheap designs, made by Messrs. Garrould, and they and the Belmont are all easy "to get up," which is a great and permanent merit.

### Recent Advances in Abdominal Surgery: Sterilised Salt Solution.

By Ada S. Graham, Gynæcological Operating Room Nurse to the Johns Hopkins Hospital. Read before the Nurses' Journal Club of the Johns Hopkins Hospital, January 11th, 1892.

Dating from Lister's introduction of antiseptic methods in surgical practice in the sixties, founded upon the views of putrefaction and fermentation which then existed, the chief source of the presence of bacteria in infected wounds was long thought to be the air; for this reason the carbolic acid spray was introduced, and the wounds were protected by elaborate layers of antiseptic dressings. The hands were also disinfected by strong antiseptics, and instruments were kept in solutions of carbolic acid or bichloride of mercury during every operation.

In abdominal operations, the greatest care was exercised to prevent possible infection from the air. An especial room was set apart for the operation, which previous to and during the operation, was sprayed with carbolic acid, and but a limited number of spectators were allowed to be present.

Great changes have been made recently in all these particulars; within the past five or six years even abdominal operations have been successfully performed in public amphitheatres, where the most critical operations have been successfully performed before hundreds of spectators, without enforcing any particular rules on their part. The results following those early antiseptic methods were good, as shown by the hospital statistical records.

In the light of our present knowledge of the causes of wound infection, however, these good results appear in a great part to be due to infrequent dressings.

Following Lister, Koch, by his bacteriological experiments with disinfectants, placed bi-chloride of mercury first on the list of germicides, stating that a solution of 1-1,000 would kill virulent anthrax spores after an exposure of one minute, and pus organisms after five minutes.

Recent experiments have again overturned this idol, for Gebhard has shown that anthrax spores were not destroyed after being exposed to solutions of the above strengths for 72 hours. Experiments recently published by Dr. Abbot from the Pathological Laboratory of the Johns Hopkins Hospital, and repeated and confirmed in the gynæcological field of Dr. Kelly, conclusively prove that one of the common pus organisms, staphylococcus pyogenes aureus, survives after exposure to a mercuric chloride solution of 1-1,000, or even 1·500 for twenty minutes and longer.

Its use is by this becoming restricted, since it is furthermore shown that the drug has an injurious effect on freshly-cut surfaces, causing a superficial coagulation, necrosis, and delayed union, as confirmed by Dr. Halsted's experiments, causing him to follow some German surgeons in abandoning the irrigation of wounds during operations. This has been termed the dry method of operating.

The use of chemical solutions in the peritoneal cavity is, for such reasons, to be strongly condemned. The danger of absorption is very great, and cases have been reported of death following operations where solutions of bi-chloride have been used to irrigate the abdominal cavity.

In such cases an ulcerative enteritis has been shown by numerous experiments by Dr. Robb to be formed when the peritoneal cavity of a dog is irrigated with solution of bi-chloride 1-20,000 to 30,000, one litre; fatal results uniformly follow. Well-marked ulcers of the intestines are found; in consequence of these results it is unsafe to employ bi-chloride in the abdominal cavity.

For some years past a simple sterilised salt solution has been used for irrigating the peritoneal cavity. It has been thus employed in the surgical clinic of the Johns Hopkins Hospital since its opening; it has also been long in use in the gynæcological department.

Physiological salt solution, commonly called normal salt solution, contains the normal percentage of chloride of sodium found in the blood. It has been used for a long time in pathological laboratories in the examination of frozen sections of tissue for microscopical study, on account of its non-dissolving action either on the blood corpuscles or tissue cells. This fact suggested its use in the abdominal cavity. It has, however, for some time been recommended in cases of exhausting hæmorrhage. That boiled water or even sterilised distilled water for irrigation within the abdominal cavity is in some degree hurtful would seem to have been demonstrated by numerous experiments on animals.

Pure distilled water, when introduced in large quantities, or in smaller quantities on successive days, into the abdominal cavity of dogs, caused death by rapid absorption, and the dissolving of the red corpuscles. Animals are still more readily killed by introducing water into the venous circulation. Dogs which are especially immune to certain forms of pus organisms (inoculated either intra-peritoneal or intravenous) are susceptible to the same bacteria after being made hydræmic when not enough water is used to produce the animal's death directly.

It is fair to assume, therefore, that the free use of distilled water in the abdominal cavity in the human being would lessen the resistance to infection, not only on account of harm to the tissue cells, but also on account of absorption.

The preparation of sterilised salt solution requires much care; if sterilised after the methods practiced in bacteriological work, necessitating an intelligent and especially a conscientious attention on the part of the nurse. She should thoroughly understand the principles which govern sterilisation, and should appreciate what is understood by sepsis, asepsis, and antisepsis. She should be accurate, never trusting to guessing the strength of solutions or the temperature of water.

The percentage of salt used approximates (as said before that of the blood, six-tenths of 1 per cent.; six grammes of chemically pure salt to a filtered litre of pure water, or preferably, of distilled water. In the Gynæcological Department of the Johns Hopkins Hospital glass flasks are used, holding a trifle more than two litres; space should be left for expansion. The salt is accurately weighed and dissolved in water, and then filtered into these flasks. The mouth of the flask is plugged with a wad of absorbing cotton, in the same manner as culture tubes. Over this a wad of cotton is placed, which overlaps the lipped mouth of the flask. This is neatly secured by several turns of a gauze bandage, which prevents the cotton plug from being forced out by the generating of the steam over the Bunsen flame. Stand a flask on a piece of fine wire sieve on the tripod, and allow it to boil; it is then immediately transferred to the Arnold steam steriliser, in which steam is being actively generated, and is kept here for half an hour.

This is carried out on two successive days, the boiling which requires about twenty minutes, and the subsequent sterilisation for half an hour. If this method is thus carefully carried out, the solution will remain indefinitely sterile, *i.e.*, free from any organisms, provided the flask be kept in a moderately dry place.

For use Dr. Kelly requires the heating of the salt solution to a temperature of 43 centigrade or from 108 to 112 F. At this temperature it acts as a strong stimulant and is of value in cases of haemorrhage, as well as during prolonged operations when the pulse becomes weakened, when its good effects at once become evident.

In this connection it is perhaps fitting also to refer to the good results obtained in cases of acute anæmia, which have been treated by intra-venous injection. Subcutaneously it has also been used, but probably acts too slowly in emergencies. With a weak heart the fluid fails to enter the general circulation. Other solutions have been recommended, but they possess no advantage over the saline solution.

It can be readily seen that a sterilised salt solution possesses undoubted advantages. The danger of infecting a patient with pathogenic bacteria is greatly lessened. It stimulates the lowered vitality of the tissues thus crippled by the loss of blood and makes them able to resist bacterial invasion.

As the use of the salt solution is becoming more and more popular in hospital work, it has seemed to me of interest to nurses to have an intelligent understanding of its application as well as the mechanical part that falls to their lot of preparing it.

### Everybody's Opinion.

### PRIVATE NURSES AND THEIR WAYS.

"BETSY PRIGG" writes: "V. M. H." is quite right, we are constantly hearing all sorts of complaints of private nurses, some of the most startling character, and of course we are sure there must be some foundation for them, but there are two sides to every question. May I, therefore, in reply to "V. M. H," ask how about the private patient's conduct, and the treatment of the private nurse? I am a private nurse, one of the offending party, and beg just to quote two instances of my own personal experience. I was sent for to a house where not many servants were kept, but enough to be comfortable; and it was in the depth of last winter, and although bitterly cold, there was but the poorest possible apology for a fire in the room where most of my time was spent. I sat and shivered, and upon one or two occasions I put on a shovel of coal, a most meagre supply of which came up once a day, but it was taken off again upon my leaving the room for a short time. The day's ménu consisted of cold breakfasts, suppers, and dinners four out of seven days of the week, partaken of in a room without a fire. I had no chance of getting warm in bed, as I lay on a couch in my patient's room, half frozen on those very cold nights. How is it possible for a nurse to be bright, happy, and cheerful under these circumstances? Yet here, in this house, I heard the various nurses they had had spoken of most disrespectfully; but I noticed they never sent a second time to the same institution. A month was the limit of my stay, and very glad I was to depart. Another of my cases was a particularly heavy one, and here again I heard the same story of strange behaviour on part of my predecessors. Of course I felt considerably scandalised, but later on, my own experience taught me, that all the fault was not on the nurses' side. I am no novice at either private or hospital nursing, and I do not hesitate to say that when a trained nurse is sent for, she should be allowed a certain amount of authority, otherwise why trouble about her being trained at all? One day, on being asked about some small matters connected with the case, I gave my opinion, but only to find myself flatly contradicted, and various people were quoted to prove how wrong I was. I carefully avoided an opinion again, and when asked to retail "some of the horrible things I had seen," I gently declined, saying it was not quite the thing to do, upon which I was told, "You are the first nurse I ever heard of who did not delight in telilng all the things of the kind." I was never left in peace; I was contradicted, doubted, and snubbed into an utter state of bewilderment, and after fourteen weeks I ventured to give my opinion as to the treatment I had received. Of course this immediately brought a torrent of rebuke and reproach upon me. No, when people have the misfortune to get a careless, inconsiderate nurse, the kindest thing they can do for the profession is to report the same to the Lady Superintendent of the institution from whence she came, or if she does not belong to one, to the doctor in attendance, who will soon replace her, and if they get a good nurse (and there are plenty of them only too willing to do their best for their patients) let her be treated with a moderate degree of kindness.

### Some Australian Experiences.

Dear Mr. Editor,—Having read with much interest your letters from Natal, India, Japan, &c., it occurred to me that our nursing Sisters may care to spend a few odd moments over a sketch of seven years' nursing in Australia by one who has done it. As I am a nurse by profession and not an author, I must claim indulgence for shortcomings, but I hope I shall succeed in showing how things are conducted fifteen thousand miles over the sea.

To begin with, I sailed out to Australia, via the Cape of Good Hope, and, after a most delightful voyage of four months, we arrived at Sydney, New South Wales, on a glorious afternoon in February; so much has been written about beautiful Sydney, by more able pens than mine, that I can only say we were all completely enchanted. With the exception of my fellow voyagers, I knew no one in this new strange land. I held two letters of introduction, one to the Governess's Home, Darlinghurst, the other to the Matron of the Nursing Institution, Phillip Street, at that time the only one in Sydney. Armed with our letters, I and a fellow traveller decided to land as soon as the ship anchored, so each of us having packed a small valise, we chartered a small boat and landed. It seemed very natural to call a "hansom," and drive off as though in London, and as we passsed the very fine buildings and substantial residences, the windows gay with flowers, there was something so familiar in the scene that we forgot to feel like "strangers in a strange land." Arrived at our destination a most cordial welcome was accorded us by the lady in charge, and in every respect there was a decidedly English air about everything, from the long flight of stone sceps leading up to the door, to the comfortable-looking inquisitive old black cat, who came to inspect the new arrivals. As supper was just in, we were shown into a nice bright, airy, dining-room, decked unsparingly with the lovely tropical flowers for which Sydney is so famous. It was not until we were shown to our bed-rooms, where we saw the pretty white beds with the mosquito nets neatly tied back with blue ribbon, that we realised we were in Australia. Our first "callers" were the mosquitos, who are gifted in a most marvellous manner for searching out a "new chum," as fresh arrivals are called out there. Although a very insignificant-looking creature, he is a terrible fellow; he alights on his victim so gently that he is not noticed at first, but once discovered he is not easily forgotten—in fact, the amount of interest one begins to feel in him and his visits is astonishing. There is still a great mystery to be solved concerning him—viz., how he contrives to get through the finest possible net. Next day being Sunday, we went to morning service at the Cathedral, which was very enjoyable. Bishop Barry preached, which struck another "home chord."

I made an early call at the Nursing Institution, and was most kindly received by the Matron, who gave me the choice of entering at once either as "staff nurse," at £52 per annum and uniform, indoor and out, or as "non-resident nurse." In the latter case you are entered on the books, "nurse under the institution rules." You pay 5s. for each case procured through the institution, but have to take the responsibility of getting the fees, which are your own, and range from two to four guineas weekly. I am pleased to record that all the time I was private nursing in Australia I did not lose a penny through non-payment, and the only people who demurred about the fee were, I regret to say, English. They could not learn, I suppose, to do in Australia as Australia does. I was entered as a "non-resident nurse," and instructed to hold myself in readiness to be called upon at any moment, and two days after received a telegram to go a short distance into the country to nurse a gentleman—a case of enteric fever. I was instructed to call at the doctor's house first as soon as I got to the place (this is not unusual, as it often happens he has paid his visit, and it will save him the trouble of paying another, a great consideration when he is hard pressed). When I reached the doctor's house he was out, but his wife kindly received me, and insisted upon my partaking of cake and cool lemon juice and water, which was most acceptable after my hot and dusty train journey. We chatted until the doctor came in, and after getting particulars of my case I proceeded to it. It was at a small cottage, just like a doll's house, and so compact. There were five rooms in it, a tiny dining and drawing room, separated by folding doors, a spare bed-room, the owner's bed-room, kitchen, and bath-room, all diminutive, but beautifully kept. I was received by the wife of my future patient, quite a young girl, who was naturally in great trouble. I found my patient on a feather bed, and this in the heat of a tropical climate! The room, as I said before, was very small, with only one window, which looked out upon a small passage between the two cottages, consequently the ventilation was doubtful and the patient very uncomfortable. I took his temperature, which was 105.2, and there was no prospect of getting any cool air into the room. So I, therefore, asked the wife if she would mind turning the drawing-room into a bed-room for a time—rather a cool request, you will say, but the case was urgent, and the best had to be done for it. She readily consented, so after making my patient as comfortable as circumstances would allow, we set to work and soon converted the drawing-room into a cosy bed-room, put in a single bedstead with hair mattress, and by the time the doctor arrived we were ready, with his permission, to remove the patient. He was very pleased, and assisted us in getting our charge into his new quarters and purer atmosphere. It was a trying case, and he was delirious by the second week. All this is nothing out of the ordinary course, you will say. Certainly not; on the contrary, it was a "good case." But what was out of the ordinary course was this, there was no servant or assistance of any description to be had. The word "fever" acts as a scare, and no one will do a thing for you. In the meantime, the patient requires frequent changes of linen, food has to be cooked for those in attendance, the house has to be kept clean, and ice and medicine have to be fetched; in fact, all the usual work of the house is added to the nurse's duties. Nurses being so scarce, a second was out of the question. So the next best thing was to work as well as we could between us. My patient's wife was most intelligent and willing to do exactly as I wished, so we managed to get along very well, better, in fact, than I've often done in houses where the servants were not too careful or obliging. We had a severe time of it, but our labours were crowned with success, and our patient did wonderfully well. I was really very happy there, and it was with sincere regret that I left sooner than I should have done; but nurses were so scarce, that the doctor asked me to take another case, more urgent, so I bid good-bye to my first case.

*(To be continued.)*

### "What the 'Register of Trained Nurses' really is."

Mr. Bonham Carter, Secretary of the Nightingale Fund, who sent us a pamphlet on "The Registration of Nurses and the R.B.N.A.," an extract from which was published, under this heading, in The Hospital, on April 23rd, desires us to state that the pamphlet in question was sent for publication under a misapprehension.

### Four Months in a Hospital Ward.

A PERSONAL EXPERIENCE IN A PROVINCIAL HOSPITAL—V.

### Golden Days.

Our golden days were Wednesdays and Sundays, when we were allowed to receive visitors, the time allotted being on Wednesdays from two o'clock until a quarter to four, and on Sundays from a quarter to two until a quarter to three. Two visitors only were allowed at the bedside of each patient, but four might halve the time, the first two leaving the ward before the others were admitted; a salutary rule, as the ward would be inconveniently crowded were no limit placed upon the numbers, to say nothing of the risk of wearying the patients, who are naturally in a state of excitement on these occasions. How eagerly we used to look forward to the arrival of our friends, and as the hour approached, those of us who commanded a view of the corridor, would keep a sharp look out and telegraph the first arrival to the rest of the ward, and we used to speculate as to who would be first up. The wife of a poor miner, named Sheldrake, who had lain sixteen weeks in the ward, was invariably the first while he was with us, and considering that she had three miles to walk and never once missed "visitors' day," she indeed proved herself a devoted wife.

### Baskets of Delicacies.

I expect this being "first up" meant a good deal of rough elbowing at the gates, for the visitors, a large number, are only admitted one at a time, in order that their baskets of "delicacies" may be searched for contraband items, and the utmost vigilance has to be exercised to prevent the introduction particularly of wines and spirits for no amount of written notices, exhortations, and warnings, avail to restrain some of the women from endeavouring to convey surreptitiously "a drop of the cratur" to their suffering mankind. Having passed the eye of the porter, the basket may be considered safe, except in certain cases, such as "typhoid fevers," over which a special guard is set of one or two probationers during the time the visitors are in.

### Pig's Chitterlings.

One woman was stopped with a jug of "pig's chitterlings" under her shawl, and was highly indignant when rebuked for such an exhibition of ignorance as to what constitutes proper food for a man down with fever. It is a rule that the patients themselves shall not give of their dainty stores to each other without the sanction of the Charge Nurse, but, excepting only in the typhoid fever and other extreme cases, I am glad to say, it was not enforced, and I never saw the unwritten privilege abused. In a couple of days the nature of the illness of a new comer is in some inscrutable manner known to every patient in the ward, and if solids are prohibited, the patients are as loyal to the rule as would be the nurses themselves. It would, indeed, be a pity to throw cold water upon the unselfish generosity I saw displayed around me.

### Please not to Cry.

Among our visitors the fair sex largely predominated, and were frequently accompanied by little children to help cheer and support the hearts of these stricken bread-winners. I think it would be well to have a notice put up outside the door, somewhat after this kind, "Please not to cry," for many a time have I seen a man who has borne up bravely all the week, break down utterly at the sight of the ill-advised tears of his friends; one patient particularly I remember who was a victim to this unwise exhibition of sorrow. He was as cheery as possible in the morning, and was looking forward with great delight to the arrival of his mother and sister. Directly they reached his bedside they burst into tears and continued to weep without cessation until the bell rang to clear the ward. Of course the man broke down, and it ended in his going home with his relatives that afternoon in a cab, just as he was beginning to benefit by the hospital *régime* and treatment. The first quarter of an hour of the visitors' time is spent in stowing away the tea, sugar, butter, eggs, fruit, books, newspapers, &c., which they have lovingly brought to their respective invalids; then there is a taking in of clean linen for the week, and then we settle down to a quiet conversation on home topics and news, each little group oblivious of all else. Loving words of counsel and of hope, with mutual exhortations to courage, and genuine heartfelt expressions of devotion are interchanged without fear of listening ears, for these people have the instinctive good breeding to respect the sacred character of the interviews taking place around them.

### The Last Quarter of an Hour.

During the last quarter of an hour, when topics are exhausted, there is a general looking round and an interchange of recognitions, as even the visitors, after a couple of weeks, seem to belong to our community, and take a general interest in the welfare of all of us, and here and there, where a patient is seen to be without visitors, one or another will supply the deficiency. The Charge Nurse now is in great request as every individual visitor wishes to hear from her own lips her opinion of the particular patient they have come to see, and not a little tact it must take to give such answers to these querists as shall be true and yet not alarm, for the information is, of course, carried straight to the patient, to whom an adverse or doubtful opinion might be as the turning of the balance against him. The bell has rung, and with slower footsteps than when entering, the visitors gradually go, some triumphantly escorting away a "discharged" patient who with instinctive politeness shakes hands with his old messmates and bids them be of good heart for he has been cured and why not they?

### Hide and Seek.

Presently the house porter comes up and emphasises the fact that the bell has rung, and the lingerers are forced to say a really final "good-bye." The miner's wife before mentioned, as invariably the first to come was likewise invariably the last to go. She would get halfway down the ward at least half-a-dozen times, and then scuttle back to her husband's bedside until it became positively ludicrous, in fact she played the porter a game of "hide and seek," until at last he generally had to come and lead her away. Upon one occasion when entertaining us in this manner, she slipped on the waxed floor and came down a tremendous bump, literally shaking the place and our sides at the same time with laughter. She took it very good humouredly though, and only wondered "what they wanted to grease them floors so much for."

### A Melancholy Half-hour.

The half-hour following the exodus of our friends was always a melancholy one, but the arrival of tea revived us, and then like a lot of big schoolboys we exhibited our "goodies" and set ourselves to look steadily forward to next visitors' day, always hoping that by then we should be able to receive our friends "sitting up," or at least have some encouraging remark from the doctor to communicate. It was on visitors' days that we used to get news of the welfare of former patients who had left us, and very remarkable indeed was this keen interest in the well-being or otherwise of old comrades in sickness; a proof to my mind of the genuine character of the friendships which owe their origin to common misfortune.

(*To be continued.*)

### A Sheep in Wolf's Clothing.

Dolly was always being teased about her "black sheep," as people called them.

And all the vindication which she deigned to afford those who spoke thus disparagingly of her friends was, "I am sure you all mean it kindly when you suggest I give 'So-and-so' up, because he or she did this thing or that thing—of course, I know you mean it well; but how often must I tell you that I cannot bring myself to cut anyone because they happen to do wild things; no I just stick by them all the same."

Some such answer it was, with variations, Dorothy Fleming returned to the many friends who concerned themselves on her behalf, no matter from what source; whether from her brother with whom she lived, or from some intimate girl acquaintance, the interference usually assumed the same form, and received the same answer.

And, perhaps, after all, Dolly's well-wishers had a right to expostulate, for it could not be denied, even by the girl herself, that round about her she had managed to collect a large circle of people, who, as she mildly put it, had "done wild things"; persons of both sexes these were, on whom society was a little harder than was this little female Don Quixote of 1892.

Dolly Fleming and her brother lived together in a flat down Kensington way; a pretty, fashionably furnished flat it was, with a stamp about it of extreme daintiness, characteristic of its owners.

Charlie had been a secretary some three or four years now, to a well-known political reformer, and was consequently away from his sister all day. This solitariness had developed a certain independence in Miss Dorothy, and this independence showed itself most strongly in the choice of her friends.

Nor did she care much how deeply she was offending society by thus defying it. Sometimes her brother would openly suggest to her that she seemed to go out of her way to pick up these slightly questionable associates. "And," went on Charlie on one particular occasion, "you're far too young, and far too pretty, Dolly. I tell you the plain truth, I don't like it at all. You are an awfully good girl, my dear, or I should have stopped it long ago."

"Stopped what in particular, Charlie?" his sister asked him. "What don't you really like?"

"Well," went on her guardian, "I object to certain people coming here; Fred Foster, for instance, and Jack Eaton, they are always coming here. They have an awfully bad name, you know, and I object to them being spoken of as my sister's friends!"

"They are your friends, dear," she corrected him. "You brought them here."

"Yes, Doll, long ago; but I should not have brought them now, now when it happens that I know some things which are very much to their discredit. It's no use arguing with a girl, but as you are under my charge, I tell you plainly, Dolly, I must, as your brother, put a stop, as I said, to the names of 'black sheep' being associated with yours."

He moved away as he spoke. Dorothy was standing by a window—the summer sunshine from without was streaming in on her face and figure. Charlie had said the truth when he had remarked on her prettiness. "It's horrid hard luck having to scold you," exclaimed the young man, in a repentant voice. "Doll, can't you see it for yourself? I do wish you would take up with some 'nice' people."

The girl laughed. "So many are ready to take up with 'nice people,' as you call them. Charlie, you see I thought I'd take up with some 'nasty' ones for a change, and give them a chance of being 'nice' again. If everyone gives a kick to those who are down, it doesn't help them to get up, does it? I want to help them, Charlie." She was speaking with genuine feeling in her voice and in her manner. "Perhaps," she went on, "It was rather conceited of me, but I thought I might influence them just a little. You see, dear, I really had a little plan in my madness after all; but if you object, and I know you do—well then—I'll——"

"Dolly, you are a brick," exclaimed her brother, interrupting her. "A regular little brick; I always said so."

And that night, as he lounged in the big library armchair, smoking his after dinner cigar, a sudden inspiration came to the young man; he had found this little brick of a sister of his a new subject for her mission.

Now, amongst the many habitués of the Fleming's Flat there was a certain Captain Armstrong, who had long been a professed admirer of Dorothy's, and on this admiration Dolly's brother looked with an all-approving eye.

It was on the subject of this same Captain Armstrong that Charlie's brain was turning now, and had received an inspiration.

What, if he were to call Dolly's attention again to this friend of theirs, under a new aspect, as a terrible reprobate, might she not, considering herself in the light of a reformer, allow Captain Armstrong's attentions to have more weight with her?

Charlie Fleming sat, half smiling, as he matured this plan, and he was pretty confident of his own success.

One day, some few weeks later on, young Fleming returned home from his work shortly before the dinner hour, to find himself an intruder into an apparently most earnest conversation between his sister and his friend. So deeply engrossed seemed the two, he almost regretted having entered.

"I am shocking Miss Fleming with my views, I am afraid," exclaimed the Captain.

"Charlie, *does* he mean what he says?" put in Dolly. "I am beginning not to believe he is exaggerating; I really think it is all too far-fetched."

The two men exchanged glances.

Dolly was standing in her favourite attitude near the window, twisting the blind cord in her hand, as she spoke. There seemed, to Charlie, to be a new light in her face, in her eyes.

"You don't believe, then, in Captain Armstrong wanting a little female guidance, like certain others have done before him, Dolly?" suggested the younger man, with deliberate intention in his speech, and leaving the room as he spoke.

"Well, I don't know," she answered gently; indeed, she hardly knew herself if she were pleased or angry at this new doubt in her mind. She bent her head and made still more knots in the cord.

"I know only too well," exclaimed the Captain rising, and standing near the window, too. "For I *do* want a little feminine guidance, only I want a particular guide. Dolly, will you lead me?"

"Then you were humbugging after all!" the girl expostulated, as the gong boomed out the dinner hour. The two were still alone, and Charlie had not returned.

"Yes, your brother and I planned how I was to win this answer. All is fair in love and war, you know. Indeed, I swear him a life's gratitude from this day forth. So don't be vexed. I think I can promise you shall not be angry long at our little plot."

"You were both horrid!" Dolly replied; but all the same, she did not remove the small white hand which Captain Armstrong held in his two stronger ones.

## Figures and Tables

**Figure f1:**
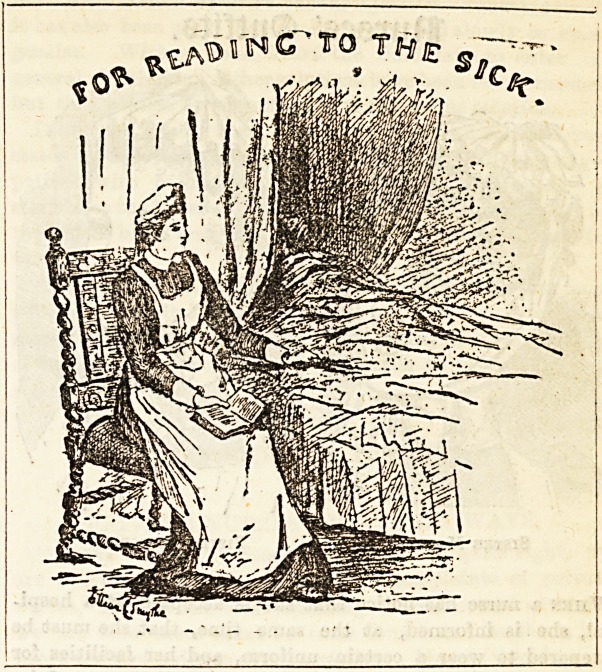


**Figure f2:**
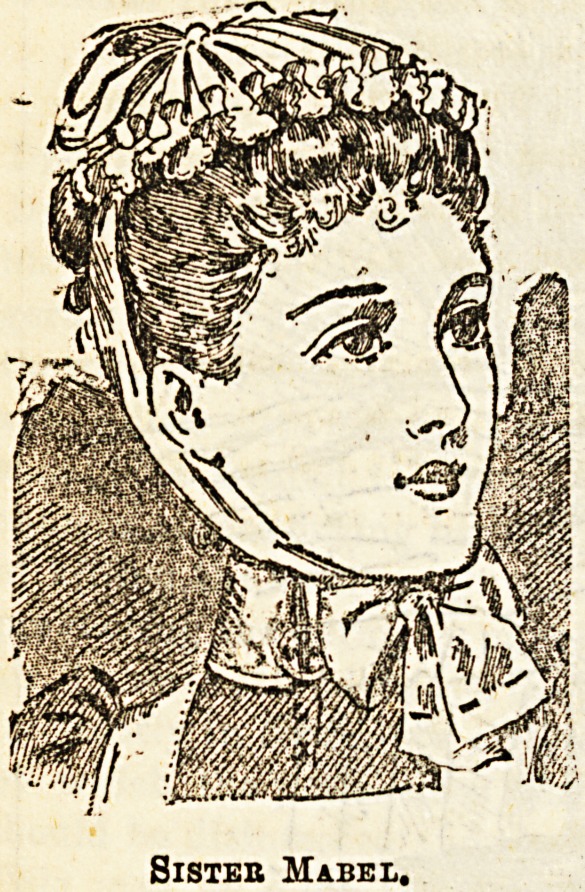


**Figure f3:**